# Athletic Sports

**Published:** 1885-01

**Authors:** 


### ATHLETIC SPORTS.

These so called hygienic exercises which have had so much attention latterly belong to the category of martyrdom and should be viewed in the light of suicide.

Among the news items of the day we observe that Dr. Amerman, of Danville, Pennsylvania, reports a case of fracture of the humerus in a base-ball pitcher, produced by muscular effort in pitching a ball to the batter, in a recent game played at that place. Other accidents of various kinds have occurred, of more or less seriousness, even to involving the life of persons, growing out of the game of base-ball.

Injuries and disabilities have resulted also from the violent exertions of those engaged in walking matches, so that it seems a fitting occasion to inquire whether the supposed advantages of these athletic sports are not more than counterbalanced by the damages sustained directly and indirectly from these extraordinary muscular efforts of the contestants.

There is a moral and social aspect of the great interest manifested in these games which allies them very closely to other species of gambling, and tends to abstract the attention of young men from the proper performance of their ordinary life-work and to demoralize their tastes for the useful and practical; but our concern is not so much with these perverted mental associations growing out of their habitual occupation in these exciting contests, as with the physical bearing of such straining and over-taxing of the powers of the muscular and bony structures of the body.

It is a well ascertained fact that a moderate and active exercise of the functions of any organ tends to develop the powers of the several components of it, and especially that regular, systematic use of the muscles imparts strength to their fibres, so that they become capable of more powerful contraction by oft repeated efforts, and are increased in bulk by the actual addition of solid tissue.

Thus it is evident that muscular exertion within certain limits develops force in the part, but when that effort is carried to the point of tension which strains the constituent elements of a muscle so as to leave it relaxed by fatigue, there is a loss of tone and consequently an impairment of the vigor in its subsequent contractions. It will be held that no such undue action is requisite in these would-be innocent pastimes, yet, practically, the spirit of high competition in these games, as in boat racing, and, we may add, prize fighting, leads to the greatest exertions of which the individuals are capable, and they are prolonged in such a manner as to tax to the uttermost every muscle of the body.

It seems to be an innate propensity of mankind to engage in enterprises which shall draw forth their utmost powers of endurance, and the recent lamentable result of another ruinous Arctic expedition is only a repetition of the same craving for self-sacrifice in useless undertakings.

If our people could only learn that their duty lay in preserving health and developing energy in their own persons while they rendered themselves useful to those around them, games of base-ball and walking matches would cease amongst us.

---

### ITEMS.

Dr. A. W. Calhoun, who was quite sick for several days in December, is out again.

The Transactions of the Medical Association of Georgia for 1884 are out. The book presents a very handsome appearance.

Dr. Thos. W. Gordon, formerly a citizen of this city, but more recently of Richmond, Va., died in McIntosh, Ga., Dec. 20th.

Dr. W. M. Fuqua, of Hopkinsville, Ky., has been recently elected to the Chair of Anatomy in the Memphis Hospital Medical College.

Dr. J. B. S. Holmes, a prominent physician of Rome, Ga., is in New York, where he will remain during the winter, in attendance upon the various clinics.

At the meeting of the Atlanta Medical and Surgical Union, held December 2d, Dr. J. D. Wilson was elected President, and Dr. J. F. Lancaster, Secretary.

Plaster of Paris dressing in the treatment of Fractures, is the title of a well written pamphlet which we have received from the author, Dr. Wm. O'Daniel, of Bullards.

Dr. L. H. Peeples, a prominent physician of South Carolina, and a graduate of the Atlanta Medical College, class of 1879, passed through the city sometime since on his way to Texas.

A child is reported as having recently died at Rye, England, from shock to the nervous system, caused by fright at seeing a boy wearing a mask. She lost her reason the first day after the occurrence.

Dr. James G. Thomas, of Savannah, died of pneumonia in Washington, D. C., Nov. 29th, where he had gone to attend a meeting of the Executive Committee of the Ninth International Medical College.

We had a pleasant call in December from Mr. F. A. Scribner, of New York, representing Messrs. Wm. Wood & Co. Mr. Scribner will make a tour in Georgia in the interest of this well known publishing house during January and February. Our readers will find him prepared to furnish them with whatever they may want in the way of medical publications.

GOLD MEDAL AWARDS TO UNITED STATES PRODUCTS AT INTERNATIONAL HEALTH EXHIBITION, LONDON, 1884.—Among the food products exhibited at the International Health Exhibition, London, 1884, from the United States, were *Beef Peptonoids* and *Maltine*; both of these preparations carried off the only Gold Medal and highest award against numerous competitors in their respective classes. All food preparations were critically analyzed at this Exhibition by a jury composed of the best chemists in the country.—*London Lancet.*

We have received from the publishers, Messrs. William Wood & Co., of New York, four volumes of Ashhurst's International Encyclopedia of Surgery. Want of space will not allow us to give this valuable work the notice which it so justly deserves; in a future number it will receive proper notice.

We had a pleasant call a few days since from Dr. T. O. Powell, Superintendent of the Insane Asylum at Milledgeville. From him we learn that there are fourteen hundred and fifty patients now in the Asylum, with over two hundred applicants who cannot be admitted for want of room.

A New York judge was annoyed by the coughing of a person in court, and after remonstrating several times in vain, exclaimed, "I am bound to stop that coughing. I'll fine you ten dollars. I think that will stop it." "Jedge," said the cadaverous man who caused the trouble, "I'd be willin' to pay twenty dollars to hev that cough stopped. Ef you ken stop it fur ten dollars, you'd better git down off'n that bench and go to practicin' medicine. There's money in it, Jedge—money in it."—*Ex.*

The following named gentlemen have been chosen officers of the International Medical Congress of 1887:

Dr. Austin Flint, Sr., of New York, President; Dr. Alfred Stille, of Philadelphia, Dr. Henry I. Bowditch, of Boston, and Dr. R. P. Howard, of Montreal, Canada, Vice-Presidents; Dr. John S. Billings, of the Army, Secretary General; Dr. J.M. Browne, of the Navy, Treasurer; and Dr. I. Minis Hays, of Philadelphia, Dr. A. Jacobi, of New York, Dr. Christopher Johnston, of Baltimore, and Dr. S. C. Busey, of Washington, members of the Executive Committee.

At a meeting of the Philadelphia Obstetrical Society, November 6th, Dr. B. F. Baer exhibited a hairpin, removed from the uterine cavity of a patient sent to him by Dr. Pancoast, of Camden, N. J. The woman, believing herself to be pregnant, had tried to produce an abortion by inserting the pin by grasping the points, and inserting the blunt end. The patient had obtained a view of the parts in a mirror placed upon the floor. The presence of the pin was readily detected by the uterine sound. He at first thought of dilating with tents, but the patient being greatly alarmed and very importunate, he used the steel dilator. One point of the pin became embedded in its descent in the tissues of the cervix, and required dissection to release it.—*Med. News.*

Dr. H. Perdue, of Barnesville, Ga , in an article in the *Medical and Surgical Reporter*, on epistaxis—how to cure it, recommends the following: "I will merely give one method, which, if laid down in any work, I have not seen, but which I regard as most important, as it has never failed me. It is the plugging the whole length of the nares with a piece of fat bacon or fat salt pork. I cut the meat long enough to plug the whole cavity from which the blood flows, and smooth so that it can be pushed in with a twist. I tie a short string to it, so that it can be pulled out when desired. I sometimes put a piece of adhesive strip across the end of the nose and meat to prevent from slipping out."

NO CURE, NO PAY.—A rather unusual case has lately been submitted to Judge Hagner in the Circuit Court at Washington, (Evening Star). This was a suit to recover from Dr. W. H. Hale one hundred dollars paid him by a colored man, named Meredith, under a guarantee of Hale that for that sum he would cure Meredith's son of pulmonary consumption, and in case he failed, he would refund the money. The boy died, and Meredith sued Hale before a justice of the peace and obtained judgment. The case was reopened, and judgment was given against Hale a second time, and he appealed. After the testimony of the parties was given, Judge Hagner affirmed the judgment below. He declined to consider the defendant's bill in bar for attending Meredith's wife, and decided the case solely as to the original claim. Judge Hagner read the heading of Dr. Hale's circular—"Hearth and Home, sworn circulation 63,000 copies monthly"—and the signed guarantee to cure the son, and said, "This guarantee was to perform an impossibility, to reverse a decree of the Almighty, and was akin to gypsying and voudooism, which says, 'I don't want your money, but as I must touch silver, I must have it before I can succeed.' The justice was right in his decision that the money should be paid back, and this court affirms that decision."—*Am. Practitioner.*

We are indebted to S. W. Beall, Sergeant Signal Corps U. S. A. at this station, for the following meteorological summary for November:

| | |
|---|---|
| Highest temperature | Nov. 2, 70 degrees |
| Lowest temperature | Nov. 25, 30.4 degrees |
| Greatest daily range of temperature | Nov. 8, 26 2 degrees |
| Least daily range of temperature | Nov. 26, 7.4 degrees |
| Mean daily range of temperature | 18.6 degrees |
| Mean daily dew point | 35.2 |
| Mean daily relative humidity | 57.2 |
| Prevailing direction of wind | N. W. |
| Total movement of wind | 6289 miles |
| Highest velocity of wind and direction | 32 miles West |
| Number of foggy days | none |
| Number of clear days | 16 |
| Number of fair days | 9 |
| Number of cloudy days | 5 |
| Number of days on which rain fell | 9 |
| Dates of frosts | 6th, 7th, 8th, 21st and 25th |

A MEDICAL FABLE.—Once upon a time a poor but humane physician was riding along a road which led by a dark forest, when he saw by the wayside a sick and miserable dog which had lain down to die. Moved with pity, he got down from his carriage, picked up the poor animal tenderly, and gave it some food and drink. Suddenly the dog vanished and he saw standing before him a beautiful fairy.

"You have saved me from a miserable doom by your compassion," she said. "Command now anything you wish and it shall be yours."

The astonished physician replied, "I am a poor man. I should like to be rich."

The fairy waved her wand and extended to him a piece of paper and a bottle filled with a dark-colored fluid. "Here," she said, "is a prescription for an Infallible Compound Hair Restorer. It will never fail, and it has been indorsed by all the leading clergymen on both continents. The world is yours! Do you wish more?"

"I am a quiet man," replied the doctor, "and little known. I should like to be famous."

"You shall be more; you shall be immortal." Waving her wand again, she presented him a small, dark and curiously-shaped instrument. "See," she exclaimed, "it is a new and unquestionably perfect pessary. It radically restores every malposition. Your name is blown into the side. Generations of suffering women and successful doctors will read and bless you. I have tried it myself," she added, blushing a little, and vanished.—*Boston Medical and Surgical Journal.*

THE BOY AND THE BONE-SETTER.—Speaking of bone setters recalls a good story which occurred in the North of Scotland, where one of them had risen to great fame and no small fortune by his skill. A country lad residing a few miles off had got his leg hurt at one of the local factories, and had been treated for some time by the local medical man without any good result. His mother, who had great faith in the neighboring bone-setter, wanted the lad to go to him, which he declined, preferring, as he said, the "reg'lar faculty." Eventually, however, his mother's persuasions prevailed, and he agreed to allow himself to be taken to see Daniel R——, the bone-setter. A bed for the invalid was extemporized on a cart, and, accompanied by his anxious mother, he was, after a rather painful journey, taken to the town where the bone-setter resided. The leg was duly examined, and it was found necessary to haul it very severely, in order, as the bone-setter said, "to get the bone in." The lad was liberal with his screams while this was going on, but eventually the bone was "got in," and he was told to go home and in a few days he would be all right and fit for his work. He was lifted upon the cart again, and, with his mother seated beside him, set off for home. "Didn't Danny do the thing well?" said the joyous old lady. 'Yes, he did, mother," said the lad, "but I was na sic a fool as gie him the sair leg!" The "reg'lar faculty" will, we have no doubt, appreciate the story.—*Whitehall Review—Courier of Medicine.*

